# An HPV-E6/E7 immunotherapy plus PD-1 checkpoint inhibition results in tumor regression and reduction in PD-L1 expression

**DOI:** 10.1186/2051-1426-3-S2-P449

**Published:** 2015-11-04

**Authors:** Adrian E Rice, Yvette Latchman, Joseph P Balint, John H Lee, Elizabeth S Gabitzsch, Frank R Jones

**Affiliations:** 1 Etubics Corporation, Seattle, WA, USA; 2 Sanford Cancer Research Center, University of South Dakota Medical School, Sioux Falls, SD, USA

## 

The prevalence of head and neck cancers in the USA is estimated to be about 370,000 and between 25% to 38% of these are human papilloma virus (HPV)-associated head and neck squamous cell carcinomas (HNSCC). We have developed a viral gene delivery platform to immunize against HPV 16 genes E6 and E7 (Ad5 [E1-, E2b-]-E6/E7) for the treatment of HPV-associated HNSCC. We tested the Ad5 [E1-, E2b-]-E6/E7 immunotherapy alone and in combination with programmed death-ligand 1 (PD-1) blockade in a murine HPV^+^ tumor model. As a single agent, Ad5 [E1-, E2b-]-E6/E7 induced HPV-E6/E7 cell-mediated immunity and resulted in the clearance of small tumors and an overall survival benefit in mice with larger established tumors. When immunotherapy was combined with PD-1 immune checkpoint blockade, an increased level of anti-tumor activity against large tumors was observed in addition to an improvement in survival. Tumor microenvironment analysis in Ad5 [E1-, E2b-]-E6/E7 treated mice revealed an increase in CD8^+^ tumor-infiltrating lymphocytes (TILs). In addition, we observed induction of suppressive mechanisms such as programmed death-ligand 1 (PD-L1) expression on tumor cells and an increase in PD-1^+^ TILs. When Ad5 [E1-, E2b-]-E6/E7 immunotherapy was combined with anti-PD-1 antibody, we observed CD8^+^ TILs at the same increased level but found that a smaller fraction of these were PD-1^+^. Furthermore, we observed a reduction in PD-L1 expression on tumor cells, providing a mechanism by which combination therapy favors tumor clearance and a rationale for pairing antigen-specific vaccines with checkpoint inhibitors in future clinical trials.

